# Reduced multiscale complexity of daily behavioral dynamics in autism spectrum disorder

**DOI:** 10.1002/pcn5.70016

**Published:** 2024-09-25

**Authors:** Toru Nakamura, Tomiki Sumiyoshi, Yoko Kamio, Hidetoshi Takahashi

**Affiliations:** ^1^ Institute for Datability Science Osaka University Osaka Japan; ^2^ Department of Preventive Intervention for Psychiatric Disorders National Center of Neurology and Psychiatry Tokyo Japan; ^3^ Institute of Education and Human Development Ochanomizu University Tokyo Japan; ^4^ Kochi Medical School Department of Child and Adolescent Psychiatry Kochi University Kochi Japan

**Keywords:** autism spectrum disorder, complexity, multiscale dynamics, physical activity

## Abstract

**Aim:**

Autism spectrum disorder (ASD) is difficult to diagnose objectively due to its heterogeneous and complex manifestations. This study aimed to objectively characterize the behavioral phenotypes of ASD children by exploring the multiscale behavioral dynamics.

**Methods:**

We applied behavioral organization (BO) and multiscale sample entropy (MSE) analyses to physical activity data collected from ASD and typically developing children, using wearable monitors in their daily life. We also examined their correlation with auditory startle response measures and clinical questionnaires, including the Social Responsiveness Scale (SRS) and the Strengths and Difficulties Questionnaire (SDQ).

**Results:**

A significant decrease in MSE at timescales longer than 6 min was observed in ASD children, suggesting decreased irregularity or unpredictability, potentially linked to repetitive behaviors or stereotyped patterns commonly observed in ASD. Additionally, an increase in MSE positively correlated with prepulse inhibition levels, indicating its relationship with sensorimotor gating. Moreover, the observed significant negative correlation with the total difficulty score of SDQ substantiates MSE's potential as an objective metric for assessing general mental health problems associated with ASD.

**Conclusion:**

Multiscale analysis enhances the understanding of ASD's behavioral dynamics, providing valuable metrics for real‐world assessments.

## Graphical abstract

This study offers valuable insights into the multiscale temporal structures of behavioral dynamics in autism spectrum disorder (ASD). Our findings revealed complex interactions between behavioral patterns, neural responses, and clinical symptoms, thereby enhancing our understanding of ASD. Moreover, these results indicate that concentrating on behavioral abnormalities could serve as an objective measure for evaluating ASD in real‐world environments.

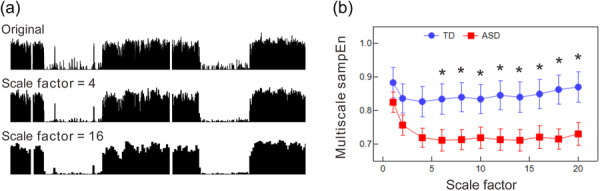

## INTRODUCTION

Autism spectrum disorder (ASD) is a neurodevelopmental condition marked by impairments in social interaction and communication, as well as by restricted, repetitive behaviors, interests, or activities.^1^ The clinical diagnosis of ASD primarily depends on identifying these symptoms. However, the complexity and heterogeneity of ASD manifestations pose significant challenges for quantification and objective diagnosis.

Evaluating physical activity has proven to be a valuable objective measure for identifying behavioral abnormalities associated with ASD. In recent studies using wearable watch‐type activity monitors,^2^, ^3^ we collected physical activity data from children diagnosed with ASD and typically developing (TD) peers in real‐world settings. These data revealed distinctive behavioral differences between the groups. Specifically, children with ASD exhibited physical activity patterns with higher mean values and greater negative skewness.^3^ These statistical features indicate hyperactivity and sporadic “troughs” of inactivity during the daytime. Additionally, these activity patterns are significantly associated with acoustic hyper‐reactivity and impaired sensorimotor gating, as demonstrated by prepulse inhibition.^3^ Furthermore, previous research found a negative correlation between daytime activity skewness and SDQ Hyperactivity Inattention scores,^2^ suggesting a link between observable activity patterns and clinical symptoms.

Despite these significant findings, the temporal structures underlying skewed physical activity patterns in children with ASD remain poorly understood. To address this gap, this study focused on the multiscale temporal structure of behavioral dynamics in ASD. We employed techniques to characterize the nonlinear dynamics of time series, such as multiscale entropy analysis^4^, ^5^, ^6^, ^7^ and behavioral organization analysis,^8^, ^9^, ^10^, ^11^ which enable a detailed dissection of patterns across various timescales that simple statistical metrics cannot capture. Through these methodologies, we aim to provide a more comprehensive understanding of the unique behavioral dynamics associated with ASD.

## MATERIALS AND METHODS

We utilized data from previous studies.^2^, ^3^ For detailed methodologies, please refer to those sources. Below, we present a brief description of the participants and the measurement data used in the study before preceding to the analysis of behavioral dynamics.

### Participants

**Figure 1:**
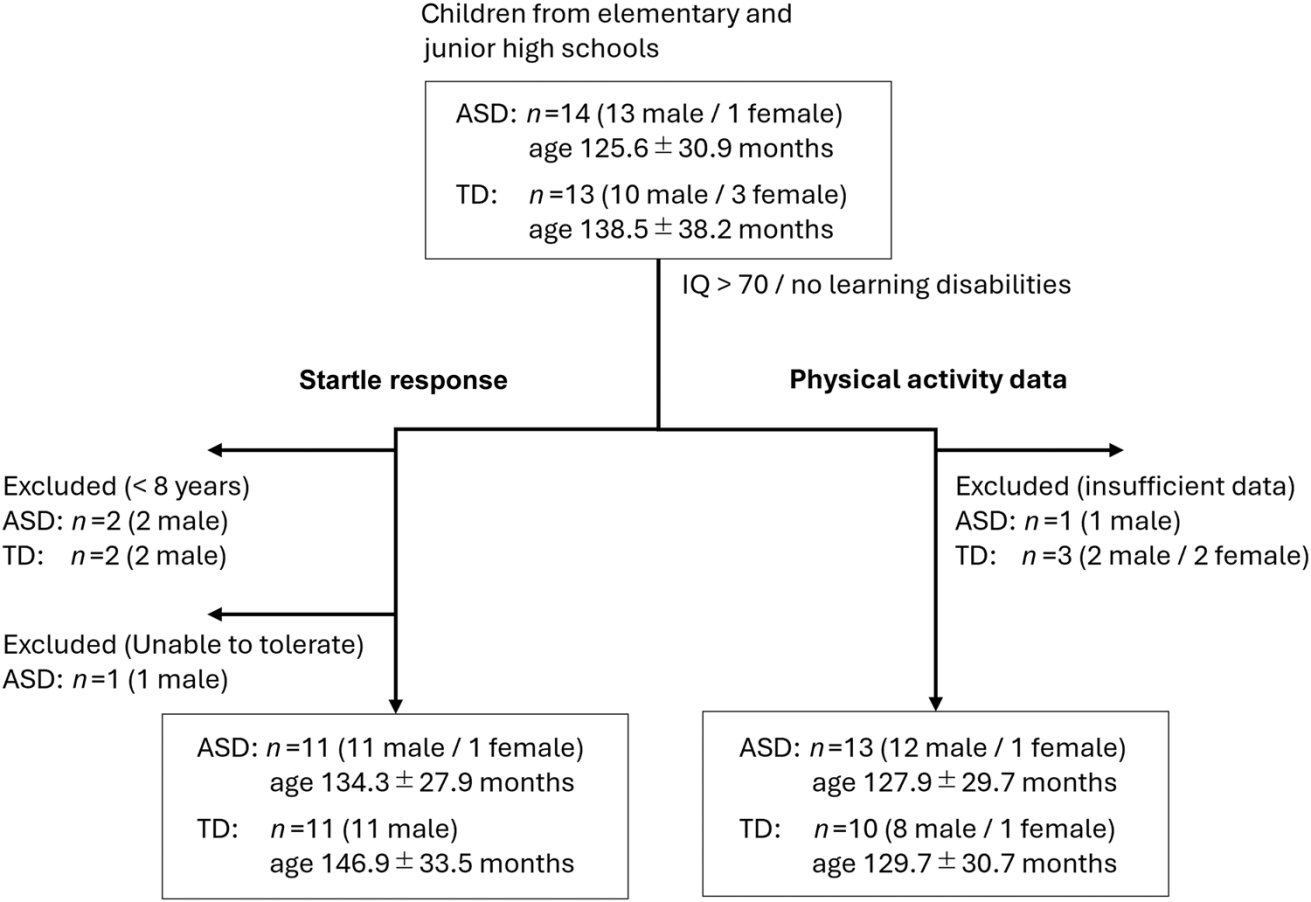
Flowchart of study population. ASD, autism spectrum disorder; TD, typically developing.

In this study, we primarily recruited children from elementary and junior high schools. A total of 27 Japanese children aged 7–16 years participated, comprising 14 with ASD (13 boys) and 13 with TD (10 boys).^2^, ^3^ Although most participants were elementary and junior high school students, a few were 16 years old due to the timing of the study during school breaks. Figure 1 shows the flowchart of the study population. Statistical analysis indicated no significant differences in sex, age (ASD: 125.6 ± 30.9 months; TD: 138.5 ± 38.2 months), or estimated IQ (ASD: 105.7 ± 23.3; TD: 104.7 ± 18.3; all above 70).^2^, ^3^ None of the children had been diagnosed with learning disabilities, had used psychotropic medications, or had a history of smoking. Children with ASD did not present with other central nervous system abnormalities, and TD children with previous psychiatric diagnoses or learning disabilities were excluded. Our sample did not include children with neurodevelopmental disorders other than ASD.

Parents assessed their children using the Japanese versions of the Social Responsiveness Scale (SRS)^12^, ^13^ to evaluate quantitative autistic traits. The SRS includes five treatment subscales: Social Awareness, Social Cognition, Social Communication, Social Motivation, and Autistic Mannerisms. Additionally, it provides two DSM‐5 compatible subscales designed to assess core aspects of autism: Social Communication and Interaction (SCI) and Restricted Interests and Repetitive Behavior (RRB). In addition, the Strengths and Difficulties Questionnaire (SDQ)^14^, ^15^ was employed to evaluate emotional and behavioral problems. The SDQ includes four difficulty subscales: Emotional Symptoms, Conduct Problems, Hyperactivity Inattention, and Peer Problems. A total difficulty score was calculated by summing the scores of these four difficulty subscales. In our previous studies,^2^, ^3^ we have detailed the scores obtained from these questionnaires. With the exception of the Emotional Symptoms and Conduct Problems subscales of the SDQ, all other scores exhibited significant group differences at the 0.05 significance level (Mann–Whitney *U*‐test).

### Ethical compliance

This study adhered to the principles of the Declaration of Helsinki and received approval from the Research Ethics Committees of the National Center of Neurology and Psychiatry (#A2013‐112). Written informed consent was obtained from all participants and their parents following a comprehensive explanation of the study procedure.

### Startle response

Startle response testing^16^, ^17^, ^18^, ^19^ was conducted to assess auditory startle response (ASR) measures, including prepulse inhibition (PPI), at various pulse intensities (65, 75, 85, 95, and 105 dB SPL).^3^ PPI measures the reduction in startle response when a weak stimulus (prepulse) precedes a stronger startle‐inducing stimulus (pulse), reflecting the brain's ability to filter out unnecessary stimuli. In our previous study, we observed a significant decrease in PPI in the ASD group at 65 dB prepulse intensity, suggesting atypical sensory processing or deficits in sensorimotor gating.^3^

During the task, participants were instructed to pay careful attention to a silent animated film to minimize boredom and reduce eye movements. The participants were continuously monitored through a 1‐way mirror, and short breaks were offered to ensure alertness and comfort during the recording session. For comprehensive details of the experiment, please refer to previous publications.^16^, ^17^, ^18^, ^19^

Because of immature neural mechanisms responsible for PPI in children under 8–10 years old,^20^, ^21^, ^22^ we excluded ASR measures from four boys (two with ASD, two with TD) who were under 8 years old. Additionally, we omitted data from one boy with ASD who could not tolerate the startle stimulus from further analysis (Figure 1).

### Physical activity data

Physical activity data were collected using a MicroMini Motionlogger actigraph (Ambulatory Monitors Inc.) with 1‐min zero‐crossing mode. Participants wore the actigraph on their nondominant wrist for more than 7 days during school vacations in spring, summer, or winter. The device was removed during bathing, rigorous exercise, or any activity that could potentially damage it. To focus on active daytime behaviors and minimize the impact of sleep durations, periods of sleep and times when the device was removed were identified using Action‐W2 software associated with the AMI actigraphic device^23^ and systematically excluded from subsequent analyses. Additionally, one boy with ASD, along with two TD boys and one TD girl, were excluded from the analysis due to insufficient daytime wear time (Figure 1).

### Evaluation of behavioral dynamics

#### Behavioral organization analysis

To quantitatively assess multiscale behavioral dynamics, particularly examining the interplay between resting and active periods in physical activity data across various time scales, we applied the behavioral organization analysis methodology developed by Nakamura et al.^9^, ^10^ This method has objectively characterized non‐trivial behavioral alterations in various clinical adult populations, including major depressive disorder,^9^ schizophrenia,^11^ bipolar disorder,^24^ and delayed sleep phase disorder.^25^ Consistent with previous studies, we calculated cumulative probability distributions P(x≥a) for durations a of both resting and active periods. These distributions were derived by numerically integrating probability density functions with a bin width of 1 min. Resting periods were defined as durations where activity counts consistently fell below a predefined threshold, while active periods were defined as durations where activity counts remained above this threshold. For this study, the threshold was determined as the overall mean of nonzero activity counts.

The cumulative distributions of resting periods were fitted with a power–law form $P(x\geq a)\;=\;Aa^{-\gamma }$, while those of active periods were modeled using a stretched exponential functional form $P(x\geq a)\;=\;\text{exp}\left(-\alpha a^{\beta }\right)$. This analysis yielded estimates for the following fitting parameters: the scaling exponent γ for resting periods, and α and β for active periods. The fitting range encompassed 2–20 min for the cumulative distributions of resting periods and 10–100 min for those of active periods.

According to Nakamura et al., a decrease in the scaling exponent γ in resting‐period distributions is associated with an increased frequency of longer resting periods in physical activity data.^9^, ^11^, ^26^ Conversely, an increase in γ corresponds to shorter resting periods.^24^, ^26^ A decrease in γ yields heightened intermittency in physical activity patterns,^9^, ^10^, ^11^, ^26^ characterized by reduced activity levels and occasional bursts. Typically, the degree of intermittency correlates closely with skewed patterns in the time‐series data. Therefore, this analysis provides deeper insight into the skewed behavioral patterns observed in ASD. Consequently, these fitting parameters serve as effective tools for exploring the underlying dynamics associated with alterations in skewness within physical activity data.

#### Multiscale sample entropy

Multiscale sample entropy expands on the concept of sample entropy by evaluating the entropy of coarse‐grained time series,^4^ thereby enabling the analysis of complexity across different temporal scales. Sample entropy measures the irregularity or unpredictability of time‐series data.^5^, ^6^, ^7^, ^27^ It quantifies the negative natural logarithm of the conditional probability that a dataset of length *N*, which has repeated itself for *m* points within a specified tolerance *r*, will continue to do so for *m* + 1 points, while excluding identical matches. For detailed algorithm descriptions, refer to the existing literature.^5^, ^7^ To ensure consistency in our analysis, we normalized the activity data to have a mean of zero and a standard deviation of 1. Following previous research, we selected *m* = 2 and *r* = 0.3 for our calculations.

We then computed the sample entropy at various scales using a coarse‐graining technique. This method involves dividing the original time series into non‐overlapping windows of length τ (scale factor) and averaging the data points within each window. This process was repeated at scale factors of 2, 4, 6, 8, 10, 12, 14, 16, 18, and 20 min, generating a coarse‐grained time series at each of these scales.

### Statistical analysis

Group comparisons of parameters from the behavioral organization analysis were conducted using the Student's *t*‐test. To assess group differences in multiscale sample entropy, a two‐factor repeated measures analysis of variance with a Bonferroni post‐hoc test was employed. Pearson's correlation was used to examine relationships between multiscale sample entropy and startle measurements, as well as clinical questionnaire scores. SAS Version 3.81 (SAS Institute) was used for these analyses. Results are presented as mean ± standard deviation, with *p*‐values less than 0.05 considered statistically significant.

## RESULTS

**Figure 2:**
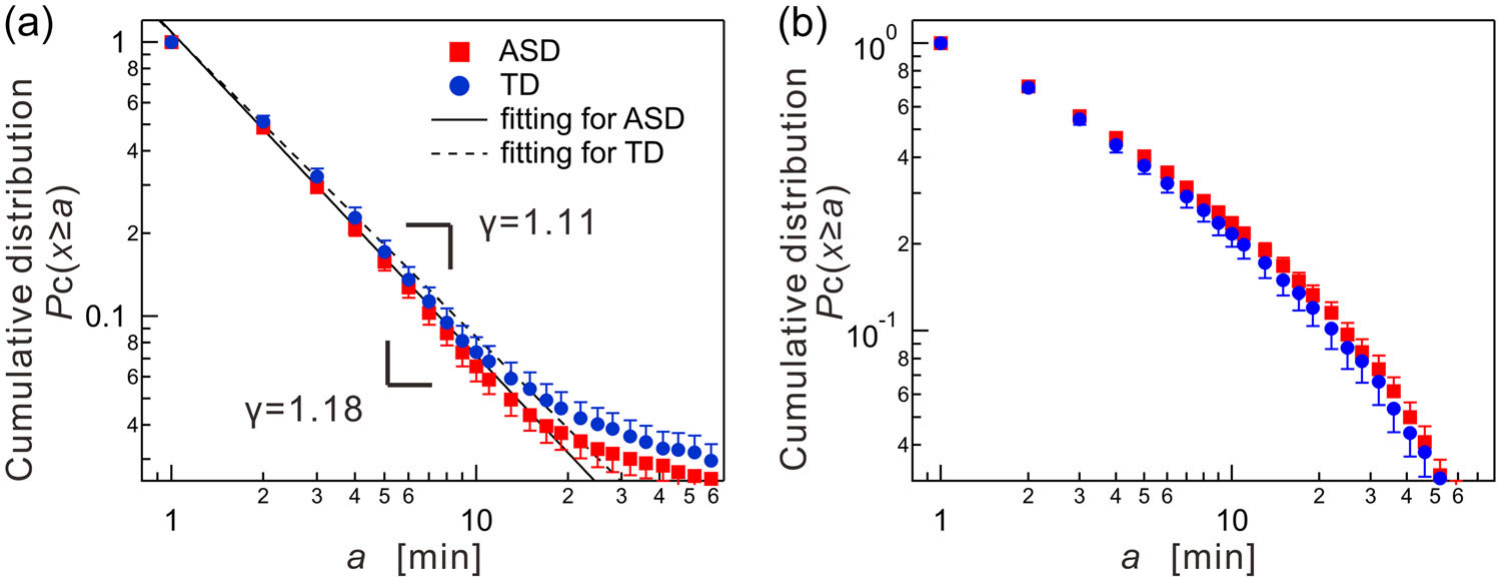
Behavioral organization analysis of daily physical activity data for typically developing (TD) children and those with autism spectrum disorder (ASD). (a) Cumulative distributions of resting‐period durations for TD (blue circles) and ASD (red rectangles) groups. Error bars show standard error of the mean. Straight lines are used as visual aids, representing the overall mean values; γ = 1.18 for ASD: γ = 1.11 TD. (b) Similar to (a), but for active period durations.

Figure 2 illustrates the average cumulative distributions of resting and active period durations for both the ASD and TD groups. Regarding resting periods, both groups displayed a power–law distribution spanning 2–20 min (Figure 2a). The ASD group consistently showed slightly lower values compared to the TD group, indicating fewer longer resting periods. Specifically, the mean scaling exponents γ were smaller for the ASD group (γ = 1.18 ± 0.17) than the TD group (γ = 1.11 ± 0.29), though this difference was not statistically significant. In contrast, the distribution of active periods for both groups followed a stretched exponential form (Figure 2b), with no significant differences observed in the fitting parameters α and β (α = 0.43 ± 0.13, β = 0.54 ± 0.08 for ASD; α = 0.51 ± 0.15, β = 0.51 ± 0.08 for TD).

**Figure 3:**
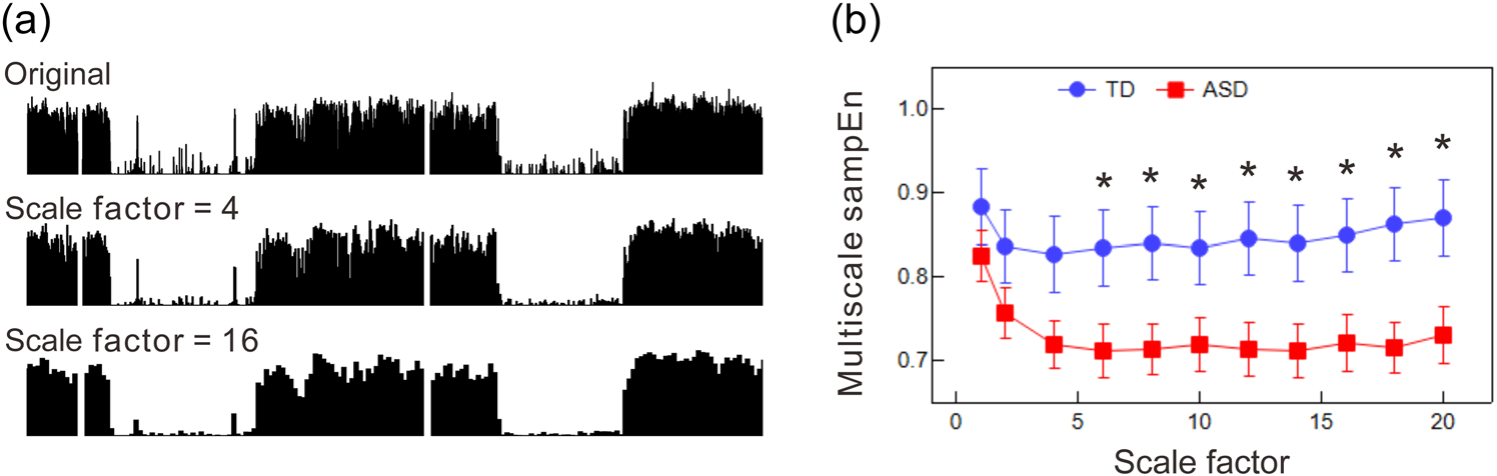
Multiscale sample entropy analysis of daily physical activity data. (a) Illustration of coarse‐graining processes. (b) Graph showing sample entropy values (sampEn) plotted against scale factor for typically developing (TD) (blue circles) and autism spectrum disorder (ASD) (red rectangles) groups. Error bars represent standard error of the mean. Asterisk (*) indicates a statistically significant group difference at the 0.05 level.

Figure 3a depicts the coarse‐grained physical activity data spanning 2 days. As the scale factor increases, micro‐fluctuations at shorter timescales are smoothed out, allowing longer timescale trends or patterns in the data to emerge. This process shifts the timescale focus of the original data, highlighting different aspects of physical activity patterns.

Figure 3b presents group comparisons of scale‐dependent changes in sample entropy derived from diurnal physical activity data. Across all the examined scale factors, the ASD group consistently exhibited lower mean entropy values compared to the TD group. Significant differences were noted at timescales greater than 6 min, indicating reduced irregularity or unpredictability in the behavioral dynamics of the ASD group. Furthermore, daytime activity data skewness demonstrated a significant positive correlation with sample entropy values at scale factors exceeding 6. For instance, Pearson's correlation coefficient was *r* = 0.42 (*p* < 0.05) at a scale factor of 6, peaking at 0.57 (*p* < 0.005) at a scale factor of 18.

**Figure 4:**
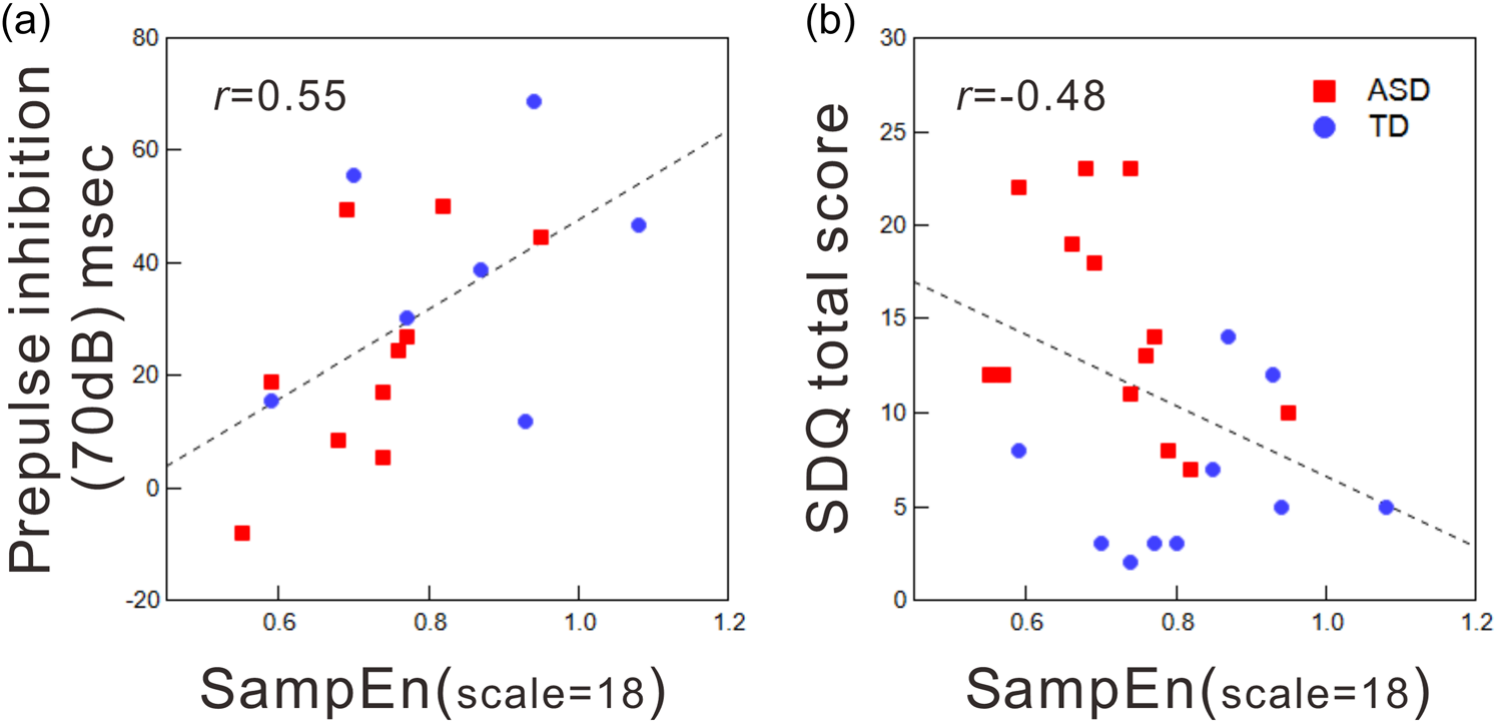
Significant correlations between multiscale sample entropy (scale factor = 18) and ASR measures and baseline questionnaires. Linear regression lines are depicted for each panel. ASD, autism spectrum disorder; SDQ, Strengths and Difficulties Questionnaire; TD, typically developing.

Figure 4 depicts correlations between sample entropy at a scale factor of 18 and ASR measures, alongside baseline questionnaires. Figure 4 shows only the relationships that were statistically significant. Among the ASR measures, only PPI at 70 dB showed a positive and significant correlation with the sample entropy (Figure 4a; Pearson's correlation coefficient *r* = 0.55, *p* < 0.05). This relationship was consistent across scale factors, ranging from 10 to 20 min. Moreover, a significant correlation was observed within the ASD group (*r* = 0.64, *p* < 0.05), but not in the TD group, when the data were analyzed separately for the TD and ASD groups.

Regarding baseline questionnaires, total difficulty scores of SDQ exhibited a negative correlation with sample entropy (Figure 4b; for example, Pearson's correlation coefficient *r* = −0.48; *p* < 0.05, entropy with a scale factor of 18). Furthermore, the SDQ subscales for Conduct Problems and Hyperactivity Inattention also showed significant negative correlations with sample entropy (*r* = −0.49; *p* < 0.05, *r* = −0.44; *p* < 0.05, respectively). This significant association was observed at scale factors between 12 and 20 min. In contrast, no significant relationship was found with the SRS.

## DISCUSSION

This study explored the multiscale structures of behavioral dynamics in TD children and those diagnosed with ASD using behavioral organization analysis and multiscale sample entropy. Our findings indicate complex interactions among behavioral patterns observed across different temporal scales, neural responses, and clinical symptoms, which traditional statistical metrics have not fully documented.

### Behavioral organization analysis

This study utilized behavioral organization analysis to explore the multiscale temporal statistical structures of resting and active behavioral dynamics in children with ASD. To our knowledge, this study is the first to apply a behavioral organization analysis to children. Importantly, our findings demonstrate that the statistical laws of behavioral organization—specifically, the power–law distribution in resting period durations and the stretched exponential distribution of active periods interwoven in the micro‐fluctuations of daily behavioral dynamics—are not only evident in adults, but also in TD children and those with ASD. This underscores the universality of these statistical features across age groups and developmental conditions.

Our analysis confirmed a trend toward an increasing γ, although this change did not reach statistical significance. This finding is significant within the context of existing research on psychiatric disorders.^8^, ^9^, ^11^ Previous research by Nakamura et al.^9^ noted that a decrease in the scaling exponent γ in the resting period distributions among adult patients with depression and schizophrenia^11^ corresponded to increased intermittency in activity patterns. These patterns are marked by lower activity levels and occasional bursts (i.e., increased positive skewness) of physical activity counts. Conversely, an increase in γ during the hypomanic phase in patients with bipolar disorder was linked with shorter durations of resting periods,^8^ indicating less stable periods of rest (i.e., attenuated intermittency associated with reduced positive skewness).

These findings indicate a potential similarity in ASD, where an increasing γ signifies a reduction in the duration and stability of resting periods. Children with ASD typically display behavioral patterns characterized by decreased downtime and increased restlessness,^1^ which may be reflected in higher γ values.

### Multiscale sample entropy

We observed distinct and consistent differences in sample entropy at longer timescales, along with a notable correlation with the simple skewness of daytime activity data. Hauge et al., without utilizing multiscale approaches, analyzed sample entropy from 1‐min actigraphic data among healthy adults, and patients with schizophrenia and major depressive disorder.^27^ They found significantly higher entropy values in the schizophrenia group compared to the depressive group, suggesting increased unpredictability or complexity, indicative of a disruption in structured daily activities. In contrast, our study demonstrated a decrease in sample entropy at timescales exceeding the 6‐min scale factor in ASD children, suggesting reduced irregularity or unpredictability.^5^ This reduction may reflect an increase in repetitive behaviors or stereotyped patterns commonly observed in ASD,^1^ or alterations in preferred behavioral patterns in specific contexts. Additionally, the negatively skewed physical activity pattern in ASD, as highlighted in our previous study,^3^ may be associated with diminished behavioral irregularity at these timescales.

Moreover, the significant correlations between sample entropy and sensorimotor gating, particularly at longer timescales with PPI at 70 dB, imply that these measures of physical activity unpredictability may reflect underlying neurophysiological processes. The reduced movement unpredictability at these scales may contribute to the findings of prior research^3^ that linked physical activity skewness to PPI.

Increased PPI and sensitization of the startle response are thought to reflect hypersensitivity to auditory information in ASD.^28^, ^29^ This neurophysiological alteration likely leads to the processing of irrelevant stimuli, resulting in the over‐filtering of subsequent sensory inputs. Such excessive auditory filtering may cause ASD children to overlook various environmental cues that prompt rich behavioral dynamics. Consequently, this may contribute to the observed decrease in behavioral unpredictability.

In contrast to earlier research^2^ that did not find significant correlations between physical activity statistics and SDQ total scores, our findings revealed a negative correlation between sample entropy and total difficulty scores of the SDQ. This correlation predominantly stems from two difficulty subscales: Conduct Problems and Hyperactivity Inattention. Notably, no significant correlations were found with the SRS, which assesses the core symptoms of ASD. These findings suggest that the observed reduced unpredictability in physical activity at longer timescales primarily reflects general mental health problems related to ASD rather than directly indicating core ASD symptoms. An alternative interpretation is that this behavioral entropy metric captures co‐occurring mental health problems related to emotional regulation in children, not limited to ASD. Further research with larger samples, including various conditions, such as attention deficit hyperactivity disorder, is needed to validate this.

#### Limitations

The primary limitation of this study is the small sample size, which may have restricted our ability to detect subtle yet potentially significant differences or associations. Additionally, the participant group consisted predominantly of boys with a wide age distribution. This demographic limitation restricts the generalizability of our findings across gender and developmental stage. Indeed, several studies have demonstrated such effects, mainly focusing on the amount of daily physical activity in children.^30^, ^31^ It is possible that these effects are also related to dynamic aspects of physical activity patterns, such as entropy. Importantly, data collection was conducted during the vacation season, which might not accurately reflect typical real‐world situations and could influence the observed behavioral patterns.

## CONCLUSIONS

This study offers valuable insights into the multiscale temporal structures of behavioral dynamics in ASD using multiscale entropy analysis and behavioral organization analysis. Our findings revealed complex interactions between behavioral patterns, neural responses, and clinical symptoms, thereby enhancing our understanding of ASD. Moreover, these results indicate that concentrating on behavioral abnormalities could serve as an objective measure for evaluating ASD in real‐world environments.

## AUTHOR CONTRIBUTIONS

H.T., T.N., and Y.K. conceived and designed the experiments. H.T., T.N., and Y.K. supervised the project. H.T. and Y.K. confirmed diagnoses. H.T. and T.N. performed the experiments. H.T., T.N., and Y.K. analyzed the data. H.T., T.N., T.S., and Y.K. wrote the manuscript. All authors read and approved the final manuscript.

## CONFLICT OF INTEREST STATEMENT

The authors declare no conflicts of interest.

## ETHICS APPROVAL STATEMENT

This study adhered to the principles of the Declaration of Helsinki and received approval from the Research Ethics Committees of the National Center of Neurology and Psychiatry (#A2013‐112).

## PATIENT CONSENT STATEMENT

Written informed consent was obtained from all participants and their parents following a comprehensive explanation of the study procedure.

## CLINICAL TRIAL REGISTRATION

N/A.

## Data Availability

The data used in this study cannot be shared publicly due to restrictions imposed by the Ethics Committee. All relevant data are included within the paper.
